# Characterization of blood flow and the effects of exogenous estradiol benzoate on residual follicles formed after ultrasound-guided transvaginal follicle aspiration in cattle

**DOI:** 10.1186/s40104-016-0117-5

**Published:** 2016-10-06

**Authors:** Alberto Mansur Ghetti, Luiz Gustavo Bruno Siqueira, Eduardo Kenji Nunes Arashiro, Miller Pereira Palhao, Felipe Zandonadi Brandao, Joao Henrique Moreira Viana

**Affiliations:** 1 Universidade Federal Fluminense, Niteroi, RJ 24230-340 Brazil; 2 Embrapa Gado de Leite, Juiz de Fora, MG 36038-330 Brazil; 3 Universidade Jose do Rosario Vellano, Alfenas, MG 37130-000 Brazil; 4 Embrapa Recursos Geneticos e Biotecnologia, PqEB Av. W5 N, Brasilia, DF 70770-917 Brazil

**Keywords:** Doppler, Ovary, Ovum pick-up, Steroidogenesis, Vascularization

## Abstract

**Background:**

Removal of the follicular content by ultrasound-guided transvaginal follicle aspiration (TVFA) may fail to induce immediate atresia and loss of function, resulting in the occurrence of residual follicles (RF). The aims of this study were to characterize the blood flow in RF and to determine the effects of the treatment with estradiol benzoate on RF fate. Lactating, cyclic Holstein-Gir crossbred cows were used. In Experiment 1, follicular wave emergence (D0) was synchronized in cows (*n* = 10) and follicular growth was then monitored by transrectal ultrasonography from D0 to D8, followed by TVFA of the largest follicle present on the ovaries 24 h later. Color Doppler ultrasound imaging was used to examine blood flow on the follicular wall, which was recorded immediately before and every 12 h after TVFA, up to 72 h. In experiment 2, cows (*n* = 22) were randomly allocated to receive either 2 mL of saline i.m. (Control group, *n* = 11) or 2 mL estradiol benzoate i.m. (EB group, *n* = 11) immediately after TVFA. Ovaries were scanned every 12 h to confirm the presence and to measure the diameter of RF. The contents of the RF, if present, were collected 72 h after the first TVFA, using the same aspiration procedures. Follicular fluid from original follicles and RF were stored at -20 °C until hormonal assays.

**Results:**

In Experiment 1, there was no reduction (*P >* 0.05) of blood flow in the remaining follicle walls after TVFA and maximum blood flow values were observed at 49.5 ± 19.7 h post-TVFA. In Experiment 2, formation of RF after TVFA was proportionally similar between Controls (5/9) and EB (5/10) cows. Also, RF diameter did not differ between groups (*P >* 0.05). Nonetheless, the content of RF from cows in the EB group had lower (*P* = 0.0004) estradiol (E2) concentration and lower (*P* = 0.0005) E2:P4 ratio compared with Controls.

**Conclusions:**

In conclusion, 1) the persistence of vascularization in the remaining follicle wall may contribute to the formation of RF after follicle aspiration, and 2) the treatment with estradiol benzoate does not prevent formation of RF, but does reduce their estradiol production.

## Background

Ultrasound-guided transvaginal follicle aspiration (TVFA), also known as ovum pick-up (OPU), has become the technique of choice to recover cumulus-oocyte complexes for *in vitro* embryo production in cattle in most countries [1]. This technique is less invasive than other approaches as laparotomy or laparoscopy and so can be used repeatedly in the same oocyte donor with low risk of sequels [2] or disturbance of animal welfare [3]. Moreover, TVFA permits the recovery of follicular fluid and granulosa cell samples for studies on the physiology of the intrafollicular microenvironment [4, 5].

Alternatively, follicle ablation by TVFA is also extensively used as a method to synchronize the emergence of ovarian follicular waves [6–8] and to reduce the detrimental effects of follicular dominance during superovulation [9–11]. These strategies are based on the assumption that aspirated follicles will undergo atresia after removal of the fluidic content and collapse. However, previous studies from our and other groups reported that the follicle wall may remain functional after being aspirated, forming a fluid-filled antrum which eventually persist as a steroidogenically-active structure [12, 13]. These were referred to as residual follicles (RF) and their occurrence is more frequent after aspiration of large follicles, i.e., follicles likely to have greater intrafollicular estradiol (E_2_) concentrations [13]. Thus, the presence of RF may disturb the expected results of any procedures performed after follicle ablation by TVFA, such as follicular wave synchronization and/or superovulation.

The mechanisms that determine the fate of aspirated follicles are not yet fully understood. Although studies with color Doppler ultrasonography suggested that blood flow is important for both follicle and corpora lutea development [14], there are no reports on the association between blood flow after TVFA and RF occurrence. Conversely, the expected increase in FSH concentrations caused by the removal of the negative feedback of estradiol and inhibin on this hormone after the aspiration of large follicles [15] could perhaps stimulate granulosa cells on the remaining in collapsed follicles, rescuing the follicle back to functionality instead of immediate atresia and eventually promoting a rebound in E_2_ production. Plasma FSH concentrations can be artificially reduced by estradiol benzoate (EB) injections, similar to procedures used for follicular wave synchronization in various timed artificial insemination protocols [16]. Therefore, the same strategy could be used to avoid an increase in FSH concentration TVFA, potentially limiting the occurrence of RF.

Thus, the aims of the this study were to characterize the blood flow in the remaining follicular wall of RF and to determine the effects of treatment with estradiol benzoate on the rate of occurrence and steroidogenic activity of RF formed after TVFA. We hypothesized that there is no reduction in the blood flow on the wall of follicles after collapse, leading to persistent function and eventually RF formation. A second hypothesis was that treatment with estradiol benzoate treatment would prevent the formation of RF after TVFA, by suppressing FSH action on granulosa cells of the follicular wall.

## Methods

### Animals and location

This study was performed at the Embrapa’s experimental field station, located in Coronel Pacheco, MG, Brazil, from March to October, 2011. Holstein-Gir crossbred (blood share of 87.5 ± 12.1 % Holstein) lactating cows (*n* = 32), 4.9 ± 2.2 years old, with regular cyclic-luteal activity and an average body condition score of 2.4 ± 0.4 (1 to 5 scale) were used in two experiments. Cows were kept in grass pastures (*Brachiaria sp*) and supplemented with corn silage, with *ad libitum* access to minerals and water.

All of the procedures using research animals were approved by Embrapa’s ethics in the use of animals committee (Protocol CEUA-CNPGL 02/2011).

### Experimental design

In experiment 1, 10 cows were used to evaluate the changes in blood flow on the follicular wall prior to and after TVFA. The formation of a fluid-filled antrum in the collapsed follicles after TVFA was referred to as RF. To standardize the size and developmental status of the follicles at aspiration, follicular wave emergence was synchronized using a protocol designed for timed artificial insemination, except that ovulation was not induced at the end of the protocol: on d 0 (D0) cows were treated with sodium cloprostenol (500 μg im, Sincrocio, Ourofino Agronegocio, Sao Paulo, SP, Brazil), estradiol benzoate (EB, 2 mg im, Sincrodiol, Ourofino) and received an intravaginal progesterone implant (1 g, Sincrogest, Ourofino), which was removed 8 d later (D8). Follicular growth was monitored by transrectal ultrasonography (MyLab 30 Gold, Esaote, Genova, Italy, with an 8.0 MHz linear-array transducer) every 24 h from D0 to D8 and the diameter of growing follicles was recorded daily. At D9, the largest follicle present on the ovaries, presumptive dominant follicle, was measured and aspirated by TVFA, with the same ultrasound device, except that a micro-convex 7.5 MHz transducer was used for TVFA, which was performed as previously described [15]. A conventional TVFA system was adapted for individual recovery of follicular fluid (FF) from targeted follicles into 1.5 mL tubes [5]. Follicular fluid was then centrifuged at 600 × g for 10 min to remove cells and cumulus-oocyte complexes and the supernatant was stored at -20 ^o^C until radioimmunoassay (RIA) analysis. Blood flow on the follicular wall was assessed by color Doppler ultrasonography and recorded immediately before and every 12 h after TVFA, for the duration of 72 h. The settings of the color Doppler were standardized during all examinations (frequency 7.5 MHz, overall gain 70 %, pulse repetition frequency 0.7 KHz, depth 6.0 cm, velocity range +0.05 to –0.05 m/s).

In experiment 2, 22 cows were used to investigate the effects of the treatment with EB on the occurrence, growth, and steroidogenic activity of RF. The same procedures described in Exp. 1 were used to determine follicles to be aspirated. Follicle aspiration and FF recovery were also performed as described in Exp. 1. Immediately after TVFA, cows were randomly allocated to receive either 2 mL of saline im (Control group, *n* = 11) or 2 mL of EB im (2 mg, Sincrodiol, EB group, *n* = 11). Ovaries were scanned every 12 h by transrectal ultrasonography to confirm the presence/absence of RF and to measure their diameter. If present, the contents of RF were collected 72 h after the first TVFA, using the same procedures described for FF collection in Exp. 1.

### Image analysis

Video records of color Doppler ultrasound exams in both experiments 1 and 2 were used to calculate the blood flow on RF. One image (a frame of the video record) of the middle section in each RF, chosen as the most representative of the actual blood flow, was used to measure the area of the follicular wall and the area of color Doppler signal within it. Calculations were performed using the ImageJ software (http://imagej.nih.gov). Vascularization of RF was defined as the ratio between the area of follicle wall and area of color Doppler signal.

### Hormonal assays

Intrafollicular concentrations of E2 and P4 in the FF and in the content of RF were determined by solid-phase I^125^ RIA, using commercial RIA kits (TKE22 Coat-A-Count Estradiol and TKPG5 Coat-A-Count Progesterone, Siemens Healthcare Diagnostics Inc., Tarrytown, NY, USA) at the Endocrinology Laboratory of the College of Veterinary Medicine and Animal Sciences, São Paulo State University (UNESP), Botucatu, SP, Brazil, following procedures described previously [13]. If necessary, samples were diluted 1:1 to 1:1,000 to fit the standard curves. Assay sensitivity was 0.02 ng/mL and 10 pg/mL for P4 and E2, respectively. Inter- and intra-assay coefficients of variation were 2.75 and 1.87 %, respectively, for P4; and 6.0 and 12.7 % for E2. Quality control was performed according to the manufacturer’s instructions, using samples of known concentrations of P4 (0.1 and 2.7 ng/mL) and E2 (26.3 and 510.0 pg/mL).

### Statistical analysis

Data were examined for normality using the Shapiro–Wilk test and transformed to natural logarithms if needed (Exp. 1: percent change in blood flow; Exp. 2: P4 concentrations in FF and E2, P4, and diameter in RF). Raw data were used to perform statistical analysis of the other endpoints. In Experiment 1, due to a large variation in the area of vascularization in both original follicles and RF, vascularization data were transformed into percent change relative to the values observed prior to TVFA (h0) and time (h) was included in the model for analysis. In Experiment 2, analysis considered the main effects of group, time (h), and their interaction. The SAS MIXED procedure with a REPEATED statement was used to account for the autocorrelation between sequential measurements (9.3 Version; SAS Institute Inc., Cary, NC, USA). If a significant main effect of hour was detected, differences among means were determined using the Least Significant Difference test. If a significant effect of group or interaction was detected, differences between groups were determined using the Student’s *t*-test. The frequency of RF formation after TVFA in experiment 2 was analyzed using the Fisher’s exact test for differences between groups. Results are described as mean ± S.E.M. A p-value of 0.05 indicated statistical significance.

## Results

### Experiment 1

**Fig. 1:**
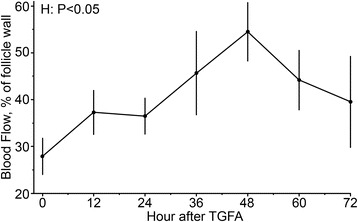
Blood flow (percentage of follicle wall showing color Doppler signal) in residual follicles at the moment (0 h) and after (12–72 h) ultrasound-guided transvaginal follicle aspiration (TVFA)

Two follicles presented E2:P4 ratio < 2 and their data were excluded from analysis. The other follicles were 13.1 ± 0.9 mm diameter at the time of TVFA and had 718.1 ± 120.4 ng/mL of E2, 42.1 ± 8.7 ng/mL of P4, and a E2:P4 ratio of 20.9 ± 5.8. Areas with positive blood flow corresponded to 27.9 ± 3.9 % of the area of follicle wall just prior to aspiration. Residual follicles were detected after TVFA in all follicles (8/8). There was no reduction (*P >* 0.05) in RF blood flow, compared with blood flow in the original follicles before TVFA. The maximum values of blood flow and blood flow percent change relative to h 0 were observed at 49.5 ± 7.0 h after TVFA; however, due to the high individual variation (84.7 %) in the maximum values of blood flow, there was no effect (*P >* 0.05) of hour on RF blood flow (Fig. 1).

### Experiment 2

**Fig. 2:**
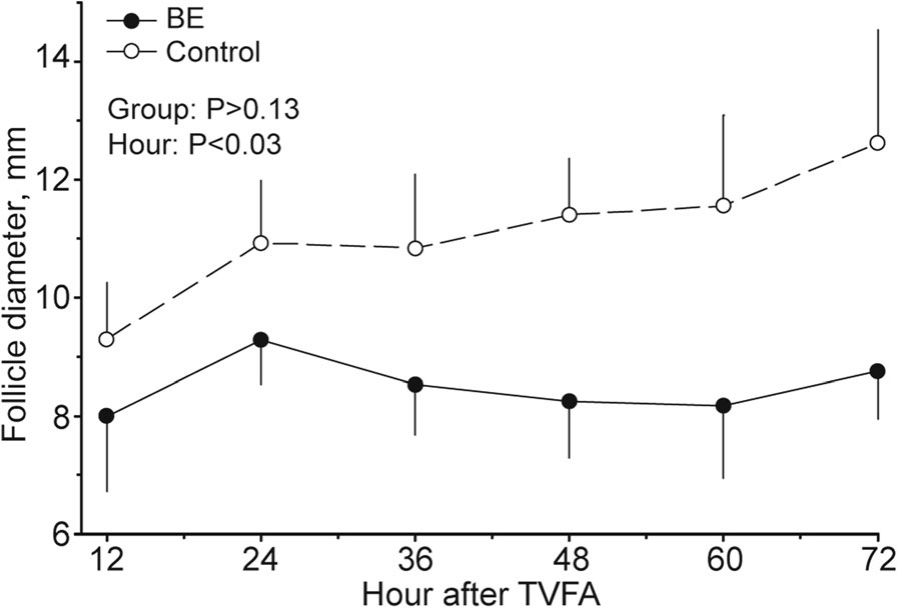
Diameter of residual follicles formed after ultrasound-guided transvaginal follicle aspiration (TVFA) in cows treated (EB group) or not (Control group) with estradiol benzoate

Data from two Control cows and one cow in the EB group were excluded because the follicles aspirated had an E2:P4 ratio < 2. There was no difference (*P >* 0.05) neither in the diameter nor in intrafollicular concentrations of E2, P4 and E2:P4 ratio between cows in the Control and EB groups (Table 1). Residual follicles were detected after aspiration in a similar (*P >* 0.05) proportion of cows in the Control (55.6 %) and EB (50.0 %) groups, but in the EB group two follicles underwent luteinization after TVFA and formed a corpus luteum-like structure. The diameter of RF did not differ between Control and BE groups 12 h after aspiration (9.3 ± 1.0 vs 8.0 ± 1.3 mm, respectively; *P >* 0.05). However, an effect of hour (*P* = 0.003) and a tendency for a group effect (*P* = 0.13) was observed up to 72 h after TVFA in the control group, perhaps caused by an increase in RF diameter in the control group. A similar pattern was not observed in the EB group (Fig. 2). In both groups, RF showed characteristics of luteinization, i.e., a decline in E2 (average 35.1-fold) and an increase in P4 concentration (average 14.7-fold), compared with the values observed in the synchronized follicles that originated the RF. In addition, E2 concentration and the E2:P4 ratio were greater in controls than in the EB group of cows (*P* = 0.04 and *P* = 0.002; respectively).

**Table 1:** Morphological and endocrine endpoints of follicles at aspiration and their subsequent residual follicles (RF) 72 h later in cows treated with estradiol benzoate (EB group) or saline (Control group) immediately after transvaginal ultrasound-guided follicle aspiration (TVFA)

Endpoint	EB	Control	*P*-value
Dominant follicles			
Diameter	12.4 ± 0.8	12.8 ± 0.9	>0.05
E2, ng/mL	958.5 ± 208.0	927.3 ± 207.8	>0.05
P4, ng/mL	71.6 ± 22.2	65.3 ± 14.3	>0.05
E2:P4	17.5 ± 5.0	15.9 ± 2.9	>0.05
Residual Follicles			
% Occurrence	50.0 % (5/10)	55.6 % (5/9)	>0.05
Diameter	8.8 ± 0.8	12.6 ± 1.9	0.01
E2, ng/mL	0.2 ± 0.1	53.5 ± 22.2	0.0004
P4, ng/mL	986.0 ± 602.9	1035.0 ± 425.9	>0.05
E2:P4 ratio, ×10^-3^	0.8 ± 0.6	51.7 ± 11.3	0.0005

E2: Estradiol-17β in follicular fluid

P4: Progesterone in follicular fluid

## Discussion

Although the formation of RF after TVFA has been described by our [13] and other groups [12, 17], little is known about the factors affecting the fate of cells comprising the follicular wall after a follicle is aspirated. Moreover, as RF may remain E2-active and therefore have potential implications for follicular dynamics, it is important to investigate strategies to control their occurrence. This study was designed to characterize the blood flow on the follicular wall of RF and to evaluate the use of estradiol benzoate as a treatment to prevent the formation of steroidogenically-active RF.

Sequential ultrasound examinations (12 h intervals) performed in experiment 1 demonstrated that RF were characterized by an initial blood clot filling the antrum 12 h after follicle aspiration, followed by a retraction and formation of a new fluid-filled cavity with an anechoic pattern. Vascularization of follicles before aspiration, calculated as the percentage of the follicle wall with color Doppler signal, had a high coefficient of variation among animals, which corroborates the findings of a previous report on large, dominant follicles in cattle [18]. In the current study, we demonstrate that there is no reduction, but actually a tendency for a transitory increase in vascularization of the follicular wall remaining after TVFA. The highest value of blood flow was observed at 49.5 ± 7.0 h after TVFA, when the sonographic aspect of RF was similar to those of normal, growing follicles, except for the presence of the retracted blood clot within its antrum. This suggest that current results were not biased by the morphological changes of collapsed follicles. The persistent vascularization on the follicular wall may have been mediated by inflammatory signals released at the needle’s puncture site, similar to that observed in other ovarian biopsy procedures [19] and to the inflammatory-like events associated with ovulation [20], which was, to some extent, mimicked as TVFA was performed close to the LH ovulatory surge [17]. Blood flow on the follicle wall was previously shown to be important for follicle development and function (steroidogenesis) [14], whereas a reduction in follicle blood flow was associated to follicular atresia [21]. Thus, we hypothesize that the preservation of blood flow on the follicular wall together with the expected rise in FSH concentrations caused by the transient decrease in plasma E2 concentrations after TVFA [22, 23], play a role in rescuing the aspirated follicle from immediate atresia back to functionality, by providing a support to E2 production by RF.

In our experimental model, we decided to use only follicles with a E2:P4 ratio > 2 and therefore data from five cows were excluded. A E2:P4 ratio of this magnitude was previously established as a criterion to determine whether a follicle is viable [24] and, thus, follicles with lower ratios are presumably undergoing atresia, as characterized by low E2 production and/or luteinization and increased P4 secretion. The concentration of E2 and the E2:P4 ratio in FF of follicles classified as viable (E2:P4 > 2) and so used in the current experiments ranged within values reported previously [24–26]. In both experiments 1 and 2, the same protocol was used to standardize the follicles to be aspirated, but interestingly the occurrence of RF after TVFA was quite different. In fact, the experimental model used (aspiration of large, E2-active follicles) was previously shown to result in a greater proportion of RF [13]. The results of experiment 2 in this study, however, suggest that the formation of RF for each individual follicle is still unpredictable after TVFA. The likelihood of the occurrence of RF after the aspiration of large, dominant follicles may have been affected by the functional status of the granulosa cells at the moment of TVFA. In this regard, further analysis of the expression of genes related to cell function, steroidogenesis, and apoptosis in the cells recovered within the follicular fluid may provide new clues to predict the fate of the punctured follicles.

The hypothesis that a treatment with EB would prevent the formation of RF was not supported by the results of experiment 2. In protocols to synchronize follicular wave emergence, EB reduced endogenous concentrations of FSH [27, 28] similarly to what occurs with endogenous estradiol under physiological conditions [29], which eventually induces follicular atresia and the emergence of a new follicular wave [27, 30]. We tested whether the same effect of EB could avoid a rise in FSH concentrations after aspiration [23] by blocking the gonadotrophic support to granulosa cell function and therefore causing immediate atresia of the remaining follicle wall. Actually, RF were observed in both Control and EB groups, which might suggest no effect of EB treatment. However, when steroidogenic activity was evaluated in the fluid content of RF, lower E2 concentrations and E2:P4 ratios where observed in the EB group, consistent with an expected loss of estrogenic function observed in dominant follicles after EB treatment [31]. Moreover, the majority of the RF in the Control group (4 of 5) increased in diameter up to 72 h after aspiration, whereas no RF in the EB group had this pattern of re-growth. Taken together, these results suggest that although EB treatment does not avoid the formation of RF, it may prevent further development of RF and reduce their E2 production.

The high P4 and low E2:P4 ratios in RF contents in both groups indicate that RF were undergoing luteinization. The absence of typical E2-active RF as previously described [13] was probably due to the greater interval from TVFA to the collection of RF fluid (72 h), which was required in this study to monitor their growth dynamics after TVFA. Thus, luteinization seems to be the eventual fate of most RF formed after the aspiration of large, E2-active follicles. In cows treated with EB, however, luteinization was perhaps hastened by the EB-induced LH surge [28, 32], as two of the aspirated follicles formed CL-like structures instead of RF. It has been demonstrated that follicles aspirated shortly after induction of the LH surge undergo luteinization and form CL-like structures [17], which are smaller than the normal CL formed after natural ovulation [33].

## Conclusions

The persistence of vascularization on the remaining follicle wall after TVFA may be one of the main factors that contribute to the maintenance of cell function in the collapsed follicle wall after TVFA, leading to the formation of RF thereafter. Moreover, treatment with EB at the moment of TVFA does not prevent the formation of RF, but reduce their estradiol production. Thus, the association of EB treatment with follicle ablation may improve synchronization results, when large, E2-active follicles are present and, consequently, likely to form RF.
